# ‘Every Run Is Hard': Endurance Athletes' Experiences of Return to Sports Participation After COVID-19—A Mixed Methods Study

**DOI:** 10.1155/2024/1506534

**Published:** 2024-11-29

**Authors:** Cheryl Anne Haley, Helena Van Aswegen, Benita Olivier

**Affiliations:** ^1^Department of Physiotherapy, Faculty of Health Sciences, University of the Witwatersrand, Johannesburg, South Africa; ^2^Department of Sport, Health Sciences and Social Work, Oxford Brookes University, Oxford, UK

**Keywords:** cardiopulmonary, COVID-19, fatigue, quality of life, return to sport

## Abstract

Protracted return to sport (RTS) following COVID-19 is common due to long-term cardiopulmonary symptoms and persistent fatigue. In athletes, low exercise tolerance may result in emotional distress. The aim of this study is to assess the symptom severity, the management thereof and the impact on quality of life (QOL) as endurance athletes return to their preillness level of sports participation. A cross-sectional survey-based mixed methods study of long-distance athletes was performed. Quantitative data included sport and disease characteristics, fatigue score and management of persistent symptoms. A total of 295 survey responses were included. The mean age was 45.1 (10.2) years and 54.7% were male. Barriers to exercise included tachycardia (72%), fatigue (72%), dyspnoea (58%) and inability to exercise at high intensities (75%). High physical and mental fatigue scores were found, the former significantly predicting return to sport. Qualitative data were collected through open-ended questions exploring challenges faced when returning to sport post-COVID-19 convalescence and the impact on their QOL. Biopsychosocial well-being constituted three themes: Physical, Psychological and Social. Categories with high code frequencies included persistent cardiopulmonary symptoms, physical fatigue, emotional distress and social disengagement. Mixing the methods revealed that the athletes' QOL deteriorated due to protracted RTS after COVID-19. A multidisciplinary approach to management may be required by endurance athletes.

## 1. Introduction

The coronavirus disease (COVID-19) has affected billions of people, including athletes who, despite the protective effect of regular exercise, may have been as frequently infected [1]. The novelty of the crisis evoked uncertainty, unpredictability and uncontrollability in the world we live in [2]. Moreover, being incapable of controlling or predicting the course of disease within one's own body may be severely psychologically taxing, especially for athletes [3]. Running is a highly accessible and affordable sport attracting long-distance adult athletes predominantly at a recreational level [4]. Cardiopulmonary fitness is so prognostic of all-cause mortality, and it is now classed as a vital sign [5]. Therefore, the impact of COVID-19 on all athletes is important, from recreational athletes enjoying the benefits of improved quality of life (QOL) to professional athletes whose careers may be defined by the slightest increments of performance [3].

Symptom severity of COVID-19 in athletic populations has been well documented [6, 7]. This multifaceted disease commonly causes mild to moderate cardiovascular symptoms, with a low prevalence of critical cardiac consequences [8]. Pulmonary limitations have been found responsible for low exercise tolerance long after COVID-19 infection, even following mild illness [7, 9]. In this study, the term long-COVID is used for new or persistent symptoms four weeks post-onset of illness as defined by the National Institute for Health and Care Excellence [10]. Long-COVID symptoms are heterogeneous, with fatigue most commonly reported, followed by dyspnoea and chest pain [11]. Endurance athletes require the ability to resist fatigue, placing a huge demand on their cardiopulmonary system [7, 12]. This symptom burden results in a protracted return to sport (RTS) and decreased general well-being [6, 13].

The virus primarily affects physical health; however, mental health consequences such as anxiety, depression and poor concentration in athletes are reported [14]. The mental health of professional athletes has gained a lot of research of late [13]. QOL is a multidimensional concept that encompasses domains related to physical, mental, social and emotional functioning [15]. It is defined by the World Health Organization (WHO) as ‘An individual's perception of their position in life in the context of the culture and value systems in which they live, and in relation to their goals, expectations, standards and concerns' [16]. There is a paucity of literature describing the post-COVID-19 biopsychosocial state of elite and recreational endurance athletes, encompassing their physical, psychological and social well-being [13].

Both quantitative and qualitative studies exist exploring the effect of the pandemic on athletes [2, 14, 17, 18]. To mitigate the spread of the virus, several major competitions were cancelled or postponed, and training facilities closed [1, 19], resulting in immediate reduction or cessation of exercise and, with it, an important mechanism for coping with stressors used by athletes [2]. Numerous studies reported the negative impacts of these measures on finances, physical activity and mental health of young elite athletes [17, 20]. However, more literature regarding the complexities of recovering from the disease itself, particularly in an endurance athlete population, is required [6, 13].

Pelliccia et al. [21] defined competitive athletes as those who exercise for six hours or more per week and recreational athletes for at least three hours, although the training load of endurance runners and cyclists is typically equal to professional athletes. Understanding the challenges faced by these athletes as they return to a high volume of cardiovascular exercise was the impetus for the study, as the results may suggest treatment strategies for athletes facing physical and mental health challenges in the future [11]. Thus, the aim of this mixed methods study is to assess the severity of COVID-19 symptoms and their management in endurance athletes and to explore the impact of the disease on their QOL as they return to preillness level of participation and performance in sport.

## 2. Methods

The researchers' positions can be described as follows: Two of the researchers are long-distance runners, and thus an insider perspective in interpreting the sport-specific data exists. None of the researchers contracted COVID-19, and thus an outsider perspective in interpreting the illness-specific data exists. All three authors are healthcare providers who understand the seriousness of the symptoms. Reflexivity exists as the researchers were aware of their own reactions that may influence their interpretation of the qualitative data [22].

### 2.1. Study Design, Setting and Participants

This is a mixed methods study with convergent design using a cross-sectional survey [23]. The quantitative data include demographic characteristics, symptom severity, management of illness, fatigue score and their approach to RTS. Understanding context was important, and thus the study elaborated on the participants' responses by collecting qualitative data through open-ended questions. The quantitative data and qualitative data were collected at the same time and given equal priority. The responses were triangulated and compared to further contextualise our understanding [23]. The Good Reporting of a Mixed Methods Study (GRAMMS) guidelines and the COREQ checklist were followed [24, 25].

**Figure 1:**
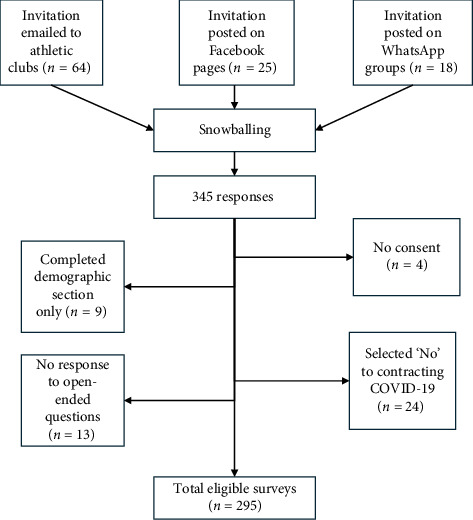
Recruitment of participants.

Adult endurance runners and cyclists of both sexes were eligible if they partook in cardiovascular exercise for three or more hours per week and were at any time diagnosed with COVID-19 by a positive polymerase chain reaction (PCR) test or experienced the known COVID-19 symptoms [7]. Athletes who were admitted to the intensive care unit (ICU) were excluded as ICU-acquired weakness occurs from 15 to 29 days and necessitates prolonged rehabilitation to return to normal function [26]. Further exclusions were not being proficient in the English language and residing outside of South Africa. Due to the nature of the study, no a priori sample size could be calculated. The recruitment of participants is illustrated in Figure 1.

Unconditional ethical clearance was provided by the University's (*information removed to allow for anonymous peer review*) Human Research Ethics Committee (Medical) (Certificate no. M21065). The study is designed according to ethical considerations in accordance with the Declaration of Helsinki, the South African Guidelines for Good Clinical Practice and the Medical Research Council. An information sheet explaining the study purpose and data sharing according to the FAIR principles [27] was linked to the survey and ensured confidentiality, anonymity and permission to withdraw from the study at any point. Due to potential COVID-19 adversary, participants were asked if they had, or would like to receive counselling.

### 2.2. Outcome Measures and Data Collection

A self-administered web-based questionnaire using the Research Electronic Data Capture (REDCap) platform was developed by the research team and based on current literature [7]. This platform was accessed through the University (*information removed to allow for anonymous peer review*). Sociodemographic data, including sex, age and sport-specific data, constituted the first ten questions, followed by eight questions on COVID-19 status, symptoms according to the NIH classification [28] and prevalence of comorbidities. The third section included questions regarding the management of their acute illness based on the WHO Severity Scale [29].

Fatigue was scored using the standardised Multidimensional Fatigue Inventory (MDFI) tool, imbedded into the questionnaire [30]. The MDFI consists of 20 questions that assess five categories of fatigue, namely, general fatigue, physical fatigue, reduced activity, reduced motivation and mental fatigue. Psychometric tests conducted on development of the MDFI showed good internal consistency (Cronbach's alpha = 0.84), construct validity established by comparing between and within groups and convergent validity analysed by correlating the MDFI score with a visual analogue scale measuring fatigue (0.22 < *r* < 0.78) [30]. In subsequent research, the scale structure of the MDFI tool was assessed using seven samples and yielded an acceptable Cronbach's alpha for most of the scales with a total score of 0.89 [31]. Although the confirmatory factor analysis could not confirm the scale structure in this study, it was advised that the MDFI structure should be maintained [31]. Our own instrument was developed by a professional panel in the field of sports medicine and respiratory health and pilot tested to identify weaknesses in the instrument. To assess content validity, three experts in the field commented on the clarity of some of the short questions and on the MDFI, a legal expert ensured anonymity and protection of personal information, two academics commented on the format of the survey and four athletes commented on the sport-specific data. The content in our study-specific questions was modified accordingly while the MDFI is a validated instrument and has not been modified for the purposes of this study. We piloted the tool among five participants who completed the survey twice, two weeks apart to avoid a learning effect. The outcome of this showed inconsistencies in the ratings of symptom severity in three surveys, so it was restructured according to the NIH symptom severity scale. One inconsistency in the impact of QOL question was found, so it remained unchanged.

The participants were given information about the objectives of the fourth section of the questionnaire, i.e., four questions exploring their RTS management, challenges faced and the psychosocial impact of this disease, followed by four open-ended questions as follows:

1. What is the main concern or challenge that you have experienced while returning to training?
2. Were you stressed about recovering fully and quickly from COVID-19?
3. Many athletes partake in sport for enjoyment, physical health, taking on a challenge and/or improved QOL. Can you comment on how COVID-19 has affected these in your life?
4. Is there any further information you can share about how COVID-19 affected you?

The primary researcher structured the questions as simply as possible to encourage a good response rate while eliciting relevant information from the athletes to answer the research question.

### 2.3. Procedures

Participants were recruited by snowball sampling from athletic clubs in South Africa between December 2021 and July 2022 [7]. Invitations to complete the online survey were disseminated using social media: 18 WhatsApp groups and 25 Facebook pages, and emailed to 64 athletic clubs. Athletic clubs approved the post on their social media pages and chats. Invitations could then be forwarded to fellow athletes by the recipients in a snowball fashion. The invitations to participate contained a link to the participant information sheet on REDCap. The survey became accessible after providing consent to participate.

### 2.4. Data Analysis

Answering ‘No' to contracting COVID-19 and absent responses to all open-ended questions resulted in exclusion from data analysis.

Quantitative data were extracted from REDCap into Microsoft Excel, cleaned and imported into IBM SPSS Statistics for Mac 28.0.1.1 (4), which was used for all analyses. Descriptive characteristics were summarised using means and standard deviations for continuous variables and percentages and frequencies for categorical variables. Regression analyses were performed using ANCOVA and multivariate logistic regression.

The qualitative data were analysed using content analysis within the deductive framework of the biopsychosocial model. The established themes included physical, psychological and social. An inductive approach to content analysis was then used where the initial codes were derived from the data itself in an exploratory analysis to allow new information to present itself about this novel disease [32]. Coded data counts indicate patterns in the data where the more commonly described codes versus less commonly described codes lie [23, 32]. This enhances the context; however, it does not attach meaning to the code [32]. Thereafter, similar codes were grouped into categories and were allocated to one of the three overarching themes. No identifiable data were extracted. Two of the authors (one athlete and one non-athlete) independently coded the responses. This was followed by a discussion to gain consensus on the codes and categories. The third author verified that the codes were allocated to the correct themes and the data sufficiently supported the themes, thereby triangulating the data and credibility of the analysis. All three authors discussed their perspective on the understanding of the data.

Mixing of data was accomplished by merging of data [23]. This integration was achieved by presenting the quantitative and qualitative results simultaneously, primarily in the discussion section. Data integration is evident in Table 1, where quantitative and qualitative data are listed side by side [23]. Additionally, Table 2 displays the content analysis revealing the frequency of citations for the codes [32].

Since the survey questions used required radio buttons, respondents had to provide an answer for each item, resulting in minimal missing data. A blank response to an open-ended question was not recorded as ‘no impact', as one cannot assume this. Participants may have felt they already noted their concerns in the first question and/or may have not thought about a particular point at the time of answering.

## 3. Results

### 3.1. Quantitative Data Analysis

Table 3 summarises the study population characteristics. The mean age was 45.1 (10.2) years, with 54.7% (*n* = 158) male respondents and 95% (*n* = 274) recreational athletes. Of those with pre-existing comorbidities (*n* = 54), 14 reported a deterioration of their condition. Based on the timeframe from onset of illness to answering the questionnaire, it can be assumed that the majority were infected with the COVID-19 beta- or delta-variant. Approximately half the athletes consulted outpatient medical professionals or were admitted to hospital.

The survey questions that focused on exercise intolerance and the management of RTS included response options suggested by the tool. These results are presented in Table 1, in the column labelled ‘Variable'. Additional information provided by the respondents in the open-ended questions that related to exercise intolerance and their RTS was coded based on content. These codes are presented in the adjacent column labelled ‘Codes related to the quantitative parameter'. The qualitative data build on the quantitative data and in this way provide more information about athletic performance post-COVID-19 [23]. Almost one-third of the athletes had not yet returned to their preillness level of sport at the time of the survey. Results of the MDFI indicate higher general fatigue and mental fatigue scores, indicating worse fatigue in these categories. The self-reported limitations to exercise indicated that athletes aimed to resume training but experienced difficulties at high loads with elevated HR and fatigue. The number of athletes who pushed through their RTS challenges closely matched those who chose to rest.

**Figure 2:**
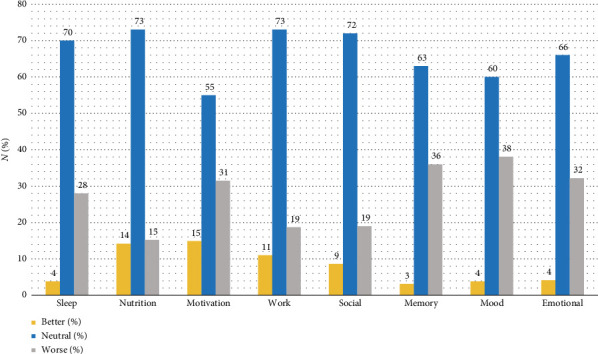
The impact of COVID-19 illness on endurance athletes' quality of life (*n* = 295).

Athletes were stressed about recovering fully and quickly from COVID-19 (63.4%), and 18% felt stigmatised due to their positive COVID-19 status. The biopsychosocial impact of their illness is illustrated in Figure 2, where the percentages of athletes citing improved, neutral or worsened qualities are displayed. Most selected a neutral response but many indicated a worsened QOL after contracting COVID-19.

In the regression analyses (Table 4), sex and severity of illness are shown to be significant determinants of fatigue; however, the low R-squared indicates that the variables in this model explain only a minor variance in the MDFI score. Severity of illness and physical fatigue were shown to be significant predictors of returning to sport in the regression model with a strong goodness of fit.

### 3.2. Qualitative Data Analysis

All responses to the open-ended questions were included in the qualitative content analysis. This generated rich data on their experiences and perceptions during their recovery from COVID-19. We identified 42 codes and 10 categories in the three key themes (physical, psychological and social) from 295 respondents (Table 2). The response rate to the open-ended questions was as follows: Question 1 was unanswered by *n* = 1; Question 2 was unanswered by *n* = 228; Question 3 was unanswered by *n* = 2; Question 4 was unanswered by *n* = 158.

### 3.3. Physical

There was a perpetual reporting of persistent cardiopulmonary symptoms well after recovering from the viral illness. The study's participants expressed concern about serious cardiac consequences following their COVID-19 convalescence. On exertion, participants emphasised a markedly elevated HR compared to pre-COVID-19 HR together with irregular HR, chest pain and longer time to return to a normal resting HR. Quantitative results showed that exercise tolerance was limited by elevated HR (72%) and dyspnoea (72%), and the severity of the initial viral illness (2%) indicating the significant fear of exercise-induced respiratory distress and having a heart attack. Only 1% of the athletes had pre-existing cardiac comorbidities, and 2.7% suffered severe illness, yet 72.2% closely monitored their HR during training while gradually increasing training time. This has negatively affected their ability to maintain a consistent pace and their confidence to train at their optimum level. Some participants required further investigations including electrocardiograms (ECGs) and blood troponin levels or medication to manage sequelae such as thrombosis, asthma and tachycardia.

*‘It's been over a year and I am still battling with breathing' [205].*

Fatigue emerged as a formidable challenge in reaching their previous fitness levels for several weeks to months after recovering from COVID-19. At the time of study, 33% of athletes had not yet reached their preillness participation level with reduced exercise tolerance the most frequently cited reason. Many participants reported low energy availability and rapid fatigue during and after physical activity. The high MDFI scores for general and mental fatigue are consistent with the burden of fatigue qualitatively described as needing to reduce exercise intensity or duration. Some individuals portrayed feeling weak, taking longer to recover from exercise and struggling with stamina and endurance. Common adaptations cited by the participants included longer complete rest compared to non-COVID-19 illnesses, listening to their bodies, adjusting their training based on physical symptoms and beginning with alternative aerobic exercise such as cycling, which is consistent with the quantitative data. The results of the multivariate analysis support these findings, with the physical fatigue scores significantly predicting the likelihood of RTS.

*‘I have had to be really patient, and accept that progress will be slow' [33].*

Ongoing postural orthostatic tachycardia syndrome (POTS) and paraesthesia are symptoms of neurodynamic dysregulation and were barriers to regaining preillness fitness. While more than half the participants monitored musculoskeletal pain in the quantitative results, only 6% cited recurrent injuries in the qualitative component. Weight gain was cited as a challenge in RTS; however, nutrition remained constant according to results in Figure 2. Altered taste and smell were deemed a nuisance and may have affected diet and nutritional intake.

*‘Fluctuating heart rate, pins and needles in my hands and body pains, especially in both Achilles tendons' [161]*

### 3.4. Psychological

In our study, the number of athletes reporting worse mood and emotional states was eight times as many as those with improved states after COVID-19. Double the number complained that they felt demotivated. Disappointment resulted as many witnessed the decline in their fitness levels. Our athletes felt a sense of purposelessness, consistent with the lost sense of normalcy and accomplishment. Many were forced to forfeit competition due to prolonged RTS, and the fear of never being able to return to their preillness level ensued. The ongoing fatigue was frustrating and athletes were not enjoying running anymore. Some cited depression and poor mental health states. These statements echo the quantitative results for motivation, mood and emotional state (Figure 2). The reduced motivation score on the MDFI supports these results. Some participants experienced devastating bereavement, grieving the death of fellow athletes, and for one, their spouse.

*‘My world has fallen apart. We use to travel the world running marathons. Now I have lost interest' [190].*

Feelings of apprehension and anxiety about their body's response and future performance capacity emanated from the lack of information about this novel virus. Anxiety or depression was pre-existing in 1.4% of the study population, yet 49.2% stated that this had become a limitation to exercise tolerance and was elucidated in the qualitative responses. According to the narrative, participants felt vulnerable and stressed about their COVID-19 positive diagnosis, fearing the unpredictable seriousness of the consequences they might suffer. The threat of critical illness propagated anxiety and prompted behavioural changes such as excessive mask-wearing, avoidance of gatherings and sanitising frequently. Considering that 70% of the participants were enrolled in the study more than three months after contracting COVID-19, the concerns of the athletes regarding persistent symptoms and protracted RTS are substantiated. The feeling that illness dominated every aspect of their lives provoked ongoing stress in their home and work lives. Evidently, exercise plays a crucial role in their resilience to occupational stress.

*‘Being unable to train and even reducing my workload post-COVID had a negative effect on my mental health and my mood in general' [125].*

Despite the severe hardships that athletes faced post-COVID-19, many athletes were not negatively impacted and few even gave positive acclamations. They were determined to resume their normal occupation, exercise routines and athletic pursuits. Positive expressions from participants included enjoying the outdoors, an increased appreciation for life, resilience in the face of adversary and reprioritised health as highly important. These findings align with the quantitative results in Figure 2, with the small percentage of participants reporting improved QOL after COVID-19. Those who experienced mild symptoms and recovered quickly expressed gratitude. Participants highlighted the role of physical activity in improving their general well-being and instilling a sense of normalcy during the pandemic.

*‘Shows that we can adapt and cope with anything that comes our way' [276].*

Mental fatigue, short-term memory deterioration and poor concentration were noted by the participants. These cognitive concerns became more pronounced when trying to balance athletic training with work or academic commitments and were exacerbated by increased anxiety. Although many did not make note of this in the open-ended questions, the quantitative component indicated that 36% experienced worsening memory after COVID-19 (Figure 2).

*‘My memory has worsened. And does not seem to be getting better either' [292]*.

### 3.5. Social

Participants faced lockdowns aimed at curbing the spread of the virus including the cancellation of races, the closure of public training facilities and the ban on group training. They expressed difficulty in adapting to new exercise routines. Additionally, exercising alone outdoors could pose safety risks. Some participants claimed the external factors of the lockdown measures had a greater impact on their lives than the illness caused by the virus. Furthermore, the restrictions had a negative impact on finances and income generated from competition. With no support from relief funds, stress in their homes and families was intensified.

*‘The lockdowns had a big impact on the social part of my running' [341].*

Many athletes cited that reduced social contact with others had a major impact on their motivation to run. Meeting friends or club mates encouraged commitment and adherence to their training. Some athletes missed the competitive element where their abilities could be tested against each other within their team or running group. Once group training was permitted, many athletes suffered from exercise intolerance and were unable to maintain the pace required to run with their team or club. Similarly, they could not participate in races as the intensity was too strenuous. Thus, the resumption of competition did not imply an immediate return to normality.

*‘I am not seeing friends I used to train with which was a huge part of my social life. Now I am having to decline because I cannot keep up with them' [135].*

Once restrictions were lifted, would gathering to regain social connection be deemed irresponsible behaviour, potentially spreading the virus, or risk contracting COVID-19 again? Society's views on appropriate behaviour during the pandemic were evident with 18.3% of athletes participating in this study feeling stigmatised. There was mention of a social stigma associated with contracting the virus, as well as the guilt of infecting others. Additionally, the COVID-19 vaccine elicited feelings of discrimination if participants chose not to be vaccinated or were precluded from vaccination due to pre-existing comorbidities. Fears of the unknown long-term consequences of the vaccination were conflicted by the benefits of vaccination in preventing critical illness and allowing attendance at sporting events.

*‘Difficult to train in groups because of the many strong disagreements people have around COVID-19/vaccines/lockdown. Less camaraderie' [87].*

## 4. Discussion

The testament of COVID-19 convalescent recreational endurance athletes has been given. Immense emphasis was placed on cardiopulmonary health after even mild or moderate illness. Most athletes were in the masters-age group and experienced increased HR and chest pain during exercise, leading to major concerns about their hearts. Prior to the COVID-19 pandemic, Kaiser-Nielsen et al. [33] suggested that due to the increasing number of recreational athletes in the masters-age group participating in high-level events, cardiac conditions may be more prevalent than in younger, elite athlete populations. Thus, the presence of serious consequences in this population should not be overlooked [7]. Our results are consistent with Juhász et al. [6], who showed that although the rate of myocardial involvement was low, nearly one-third of athletes were hindered in their RTS by persistent symptoms. The athletes adapted their RTS by pacing themselves and monitoring their fatigue. Subjective fatigue affects performance capacity, pain, emotions (reduced motivation) and social well-being [15]. Clearly, recovering from COVID-19 and returning to preillness fitness levels can be a slow and challenging process. Due to their protracted RTS, the activity they previously enjoyed was now tiresome and unappealing.

In our study, participants expressed a range of emotional and psychological difficulties in response to their inability to train and compete, which are a source of motivation for their athletic pursuits. Early in the pandemic, the paucity of information about COVID-19 made it difficult to manage individual expectations. The prolonged recovery propagates self-doubt in one's abilities, and loss of self-worth and athletic identity.2, 10 Prior to the pandemic, a review of elite athletes found alarming rates of depression, stress and anxiety despite the well-documented benefits of physical activity [13]. Exercise has positive effects on mental health by stimulating the production of endorphins in the brain, helping this study's participants cope with the stress and anxiety caused by the pandemic [20]. Although the amount of exercise required to improve mood is uncertain, increased physical activity is associated with reduced depression, anxiety and fear, regardless of age [30]. However, many had not returned to their preillness level of participation and may not have been protected from depression by their preillness resilience [35]. The participants in this study valued the enjoyment sport brings and the opportunity to achieve ambitious goals. Concerns about long-term consequences can be interpreted as an athlete's desire to be proactive in their recovery, trusting their healthcare providers. One published account of athletes' resilience to hardships focused on secondary gains such as more time for relationships and education, during imposed lockdowns [14].

Many athletes felt the absence of the social connections made through sport. The friendships made during training together were a great source of social support, which the participants longed for during the periods of lockdown. Athletes enjoyed many other benefits of training in a group such as the commitment, structure, motivation, encouragement, advice shared and general conversation. Athletes who receive social support from coaches and fellow athletes tend to have better coping mechanisms [14]. Group training in South Africa is not only attractive for the social aspect but also a profound safety measure. In the quantitative section of the survey, social factors were limited to participants' rating of their work and social life, along with the percentage reporting feeling stigmatised. The qualitative data further explored this theme. At the advent of the pandemic, avoidance of PCR tests or admitting their infection status was common due to guilty feelings and fear of social stigma [32]. There is a further stigma associated with mental health treatment due to a culture of toughness inherent in sport, and thus athletes may not acknowledge their emotional distress [36]. These sentiments imply social pressure and judgements that affect athletes. Hence, some athletes pushed themselves to train through the challenges they were experiencing. Vaccine prejudice became prominent when mass participation events resumed, and proof of vaccination was compulsory. These findings provide valuable insight into the experiences related to COVID-19 illness and the diversity of challenges that endurance athletes face in RTS.

### 4.1. Strengths and Limitations of the Study

Limitations of this study include recall bias as many of the participants' responses rely on retrospective recall and based on the participants' perceptions of threats and concerns. No response rate could be determined, reducing the power of the study. The period from illness to the time of the study varied between participants. Athletes taking part in this study would have been in various stages of the disease and infected by the range of viral strains from the more severe beta and delta to milder omicron variants. Therein lies a strength of the study; narratives from a range of temporal points and variants are included regarding this novel virus. The sample size is large. It has been found that subjective measures were superior to objective measures in detecting changes in the well-being of athletes as the training load increased [37]. Employing a mixed methods study design increases the external validity of the findings. The categories and codes were analysed by inductive coding, allowing multiple viewpoints about the impact of this novel virus. However, the validity of our own instrument is a limitation. Further investigations such as interviews and focus groups would have provided additional qualitative data to increase meaning and thereby our understanding of the real-life impact of COVID-19 on this population. The themes were deduced by the aim of the study; however, the social factors were underrecognised in the quantitative questions. This is a potential weakness of this study and further studies including quantitative analysis of social factors in this athlete population are recommended. Because the impact on RTS was interpreted from an insider perspective, personal experiences or assumptions potentially led to bias by overemphasizing certain themes.

## 5. Conclusion

This mixed methods study aimed to assess the severity and management of COVID-19 symptoms and QOL as endurance athletes returned to their preillness level of sports participation. The quantitative results found that one-third of the athletes had not returned to their preillness level of sport and complained of high HR, dyspnoea, fatigue and poor exercise tolerance. Physical fatigue scores were high and likely to explain the poor RTS rate. The qualitative results revealed testimonies of cardiopulmonary consequences, severe illness, persistent exertional symptoms, emotional distress, societal pressures and individual resilience influencing their QOL. The integration of the data found that QOL of endurance athletes deteriorated post-COVID-19 due to protracted resumption of preillness level of sport. It can be concluded that recognition of the biopsychosocial well-being of convalescent athletes is essential. A multidisciplinary approach may be required to ensure resumption of physical activity and the mental gains thereof. Lest we forget the devastating effects of the COVID-19 pandemic, future research should employ the biopsychosocial perspective to enhance athlete recovery after future illnesses.

### 5.1. Perspective

Lessons learnt from this study can be applied to future situations that result in a long-term sport hiatus, whether due to illness or injury, pandemics or political situations. A review by Williams and Hull [38] on respiratory complications post-COVID-19 suggested a need for research involving more diverse age and racial groups, which is somewhat fulfilled by this study of South African endurance athletes. This study suggests that cardiopulmonary consequences of COVID-19 should not be overlooked in the masters-age group. Both short and long-term interdisciplinary interventions are required to manage biopsychosocial well-being post-COVID-19 as the importance of resuming sport exceeds the recreational value. Pandemic-specific coping strategies and training programs should be established [39] that consider the severity of the initial illness, sex, fatigue and endurance-type sport as suggested by our results. Symptoms hindering RTS still abound after the milder omicron variant infection [7]. Athletes who received guidelines and trusted doctors were more positive, highlighting the importance of communication between athletes, coaches and primary healthcare providers. Through this understanding of perceived biopsychosocial challenges, increased confidence has been achieved leading to better outcomes in athlete rehabilitation during future health challenges.

## Figures and Tables

**Table 1:** Athletic performance-related factors (*n* = 295).

**Parameter**	**Variable**	***n* (%)**	**Codes related to the quantitative parameter**

Received RTS guidelines	Yes No	120 (40.7%) 175 (59.3%)	Followed RTS guidelines from doctor

Returned to preillness level of sport	Yes No	199 (67.5%) 96 (32.5%)	Exercise intolerance

Self-reported factors limiting exercise tolerancea	Unable to sustain intensity Difficulty hill climbing Elevated heart rate Fatigue while training Poor endurance Musculoskeletal pain Long-COVID concerns Dyspnoea Anxiety/depression Autonomic dysfunction Chest pain	221 (74.9%) 218 (73.9%) 213 (72.2%) 213 (72.2%) 198 (67.1%) 186 (63.1%) 183 (62%) 172 (58.3%) 145 (49.2%) 130 (44.1%) 64 (21.7%)	Exercise intolerance Weakness Low energy availability/fatigue Recurrent injury Tachycardia Chest pain Dyspnoea Anxiety about having a heart attack Sympathetic nervous system dysregulation Feeling anxious

Monitor during RTSa	Resting, average and maximal heart rate Pace/speed Musculoskeletal pain Fatigue Sleep quality Rate of perceived exertion Stress	213 (72.2%) 184 (62.4%) 128 (43.4%) 118 (40%) 102 (34.6%) 99 (33.6%) 52 (17.6%)	Tachycardia Recurrent injury Low energy availability/fatigue Feeling anxious Work-related stress Feeling vulnerable Sleep disturbances

Management of concerns and challenges^a^	Reduced exercise intensity Reduced exercise duration Followed RTS guidelines Resting Pushing through the challenges Cleared for exercise by a medical professional ECG Required medication Spirometry Chest X-ray/CT scan Physiotherapy	144 (48.8%) 126 (42.7%) 117 (39.7%) 117 (39.7%) 113 (38.3%) 49 (16.6%) 30 (10.2%) 19 (6.4%) 14 (4.7%) 2 (0.7%) 2 (0.7%)	RTS adaptations by pacing and increasing recovery RTS guidelines Positive influence Health awareness

**Parameter**	**Variable**	**Median (95% CI)**	**Corresponding Code**

Multidimensional Fatigue Inventory (MDFI) score	General fatigue Physical fatigue Reduced activity Reduced motivation Mental fatigue MDFI total	11.0 (10.7–11.6) 9.0 (9.1–10.1) 8.0 (8.1–8.8) 8.0 (8.2–9) 10.0 (9.7–10.7) 47.0 (46.1–49.8)	RTS adaptations by pacing and increasing recovery demotivation Frustration depression disappointment brain fog

*Note:* Data can fall into more than one category.

^a^The participants could choose more than one response.

**Table 2:** Results of the content analysis of the responses to the open-ended questions.

Themes	Categories	Codes	*n *=* *(%)	Quotation (participant number)
Physical	Persistent cardiopulmonary symptoms	Anxiety about having a heart attack Tachycardia Shortness of breath Chest pain Followed RTS guidelines from doctor Severe illness Interventions Thrombosis	60 (20.3%) 52 (17.6%) 44 (15%) 14 (4.8%) 7 (2.4%) 5 (1.7%) 2 (0.7%) 2 (0.7%)	“*I worry about the sustainability of my high heart rate during hard training. What long-tern damage am I doing due to COVID?*” *[226]*
	Physical fatigue	Exercise intolerance Low energy availability/fatigue Protracted RTS RTS adaptations by pacing and increasing recovery Weakness	95 (32.2%) 45 (15.3%) 44 (14.9%) 42 (14.2%) 23 (7.8%)	“*It's been a long road, difficult to get back to a normal level of training. Long recovery time*” *[187]*
	Neuromusculoskeletal concerns	Sympathetic nervous system dysregulation Recurrent injury Body weight fluctuations Persistent symptoms, e.g., headache	19 (6.4%) 17 (5.8%) 15 (5.1%) 11 (3.7%)	*“I also get dizzy spells one year after having COVID*” *[206]*

Psychological	Emotional distress	Disappointment Demotivation Depression Frustration Bereavement	48 (16.3%) 42 (14.2%) 28 (9.5%) 16 (5.4%) 4 (1.4%)	*“I used to run and enjoy it. Now I have to force myself to do it knowing that I will only be disappointed with my effort” [31]*
	Anxiety and fear	Feeling anxious Uncertainty Rapid loss of fitness Worried about future infections Work-related stress Feeling vulnerable Sleep disturbances	24 (8.1%) 19 (6.4%) 19 (6.4%) 8 (6.1%) 13 (4.4%) 6 (2%) 2 (0.7%)	*“It's frustrating because I feel it's all I talk about in relation to exercise. Running now adds to my anxiety rather than helps me with it” [164]*
	Positivity	No negative impact Positive influence Health awareness	60 (20.3%) 22 (7.5) 16 (5.4%)	*“The pandemic has made me aware of how blessed I am to be able to do the things I do on a daily basis” [122]*
	Cognitive concerns	Concentration Memory	7 (2.4%) 5 (1.7%)	*“I found that I had to really pull back on my physical training while writing up my thesis otherwise I could not get anything done. Terrible concentration and attention span” [285]*

Social	Societal restrictions	Lack of competition Disrupted routine Training facilities closed Financial gains of competition	27 (9.2%) 12 (4.1%) 7 (2.4%) 2 (0.7%)	*“Having an entire race season cancelled was very difficult as racing is one of my main motivations” [40]*
	Social disengagement	Missed their friends and social connections Challenging training alone as group training is prohibited	40 (13.6%) 25 (8.5%)	*“I really missed the social connections. Social interactions contribute to my motivation to get out there” [236]*
	Prejudice	Stigmatise Guilt	11 (3.7%) 3 (1%)	*“People in my running group seem nervous around me. Like I can still give them COVID-19” [65]*

**Table 3:** Summary of the study population characteristics (*n* = 295).

Characteristic	Variable	*n* (%)
Sex	Male	158 (54.7%)
	Female	131 (45.3%)

Age groups	18–29	22 (7.5%)
	30–39	68 (23.1%)
	40–49	109 (36.9%)
	50–59	77 (26.1%)
	60–71	19 (6.4%)

Type of sporta	Running	116 (39.3%)
	Cycling	52 (17.6%)
	Multisport	127 (43.1%)

Level of participation	Recreational	184 (62.4%)
	Competitive	96 (32.5%)
	Elite	15 (5.1%)

Comorbidities	Yes	
	• Asthma	17 (5.8%)
	• Hypertension	12 (4.1%)
	• Anxiety/depression	4 (1.4%)
	• Cardiac disease	3 (1%)
	• Diabetes	3 (1%)
	• Hyperthyroidism	2 (0.7%)
	• Hyperlipidaemia	1 (0.3%)
	• Other	12 (4.1%)
	None	241 (81.7%)

Time from onset of COVID-19 diseaseb	≤ 3 weeks	36 (12.2%)
	4–8 weeks	51 (17.3%)
	3–6 months	81 (27.5%)
	> 7 months	127 (43%)

Severity of COVID-19 illness^c^	Mild	196 (66.4%)
	Moderate (dyspnoea SpO2 ≥ 94%)	91 (30.9%)
	Severe (dyspnoea SpO2 < 94%)	8 (2.7%)

Treatment	WHO severityd = 0–2: no treatment or did not consult a healthcare professional	148 (50.2%)
	WHO severity = 3: consulted a healthcare professional or admitted to hospital	147 (49.8%)

^a^‘Running' = running only, ‘cycling' = cycling only and ‘multisport' = running and/or cycling and other sports.

^b^We assume variant based on the elapsed time since onset of illness: omicron < 8 weeks, beta 3–6 months and delta > 7 months.

^c^NIH symptom classification: mild: fatigue; malaise; headache; myalgia; ageusia/anosmia; cough; fever; diarrhoea; moderate: dyspnoea with SpO2 ≥ 94; severe: dyspnoea with SpO2 < 94.

^d^World Health Organization COVID-19 severity scale.

**Table 4:** Regression analyses.

**The relationship of variables with the total Multidimensional Fatigue Inventory score (R-square = 0.088, lack of fit test** **p**=0.742**)**			
**Correlations**	***B***	**95% CI**	**p** **value**

Sex	7.439	3.763–11.116	< 0.001⁣^∗^
Age	0.092	−0.089–0.273	0.317
Level	1.204	−1.852–4.260	0.439
Severity of illness	6.296	2.476–10.115	0.001⁣^∗^
Stage of disease	−0.343	−1.577–0.891	0.585

**Multivariate logistic regression analysis to determine the likelihood of variables explaining RTS (Hosmer and Lemeshow goodness of fit test** **p**=0.713**)**			
	**OR**	**95% CI**	**p** **value**

Age	1.015	0.985–1.047	0.329
Sex	1.501	0.795–2.833	0.211
Severe illness	7.195	1.184–43.724	0.032⁣^∗^
Physical fatigue	0.688	0.606–0.782	< 0.001⁣^∗^
Reduced activity	0.891	0.765–1.039	0.141
Mental fatigue	1.091	0.990–1.203	0.079
Reduced motivation	1.029	0.893–1.186	0.690

⁣^∗^significance level is *p* < 0.05.

## Data Availability

The data that support the findings of this study are available from the corresponding author upon reasonable request.
